# Molecular characterization of virulence and multidrug resistance in *Escherichia coli* isolated from houseflies (*Musca domestica*) at food markets in Northern Thailand

**DOI:** 10.14202/vetworld.2026.714-724

**Published:** 2026-02-26

**Authors:** Krissana Khoothiam, Sorawit Upakut, Achiraya Siriphap, Ornampai Japa, Chutamas Thepmalee, Nittiya Suwannasom

**Affiliations:** 1 Division of Microbiology, School of Medical Sciences, University of Phayao, Phayao 56000, Thailand; 2 Division of Biochemistry, School of Medical Sciences, University of Phayao, Phayao 56000, Thailand

**Keywords:** antimicrobial resistance, *Escherichia coli*, food markets, houseflies, multidrug resistance, Northern Thailand, One Health, virulence genes

## Abstract

**Background and Aim:**

Houseflies (*Musca domestica*) are recognized mechanical vectors of foodborne pathogens and antimicrobial-resistant bacteria, particularly in environments with intensive food-handling. Their role in disseminating virulent and multidrug-resistant (MDR) *Escherichia coli* at the food–environment–human interface remains underexplored in Northern Thailand. This study aimed to determine the prevalence, phylogenetic distribution, virulence gene carriage, antimicrobial resistance profiles, and resistance determinants of *E. coli* isolated from houseflies collected at food markets in Phayao Province.

**Materials and Methods:**

A cross-sectional surveillance study was conducted between June and November 2023 in Muang District, Phayao Province. A total of 350 houseflies were collected from meat, vegetable, and cooked-food markets using sticky traps. *E. coli* isolates were identified by culture and polymerase chain reaction (PCR) targeting the *uidA* gene. Phylogenetic grouping was performed using *chuA*, *yjaA*, and *TSPE4.C2*. Virulence genes associated with diarrheagenic *E. coli* were detected by PCR. Antimicrobial susceptibility testing was conducted using the disc diffusion method against 12 antibiotics representing eight antimicrobial classes. Multidrug resistance was defined as resistance to three or more antimicrobial classes. Resistance-associated genes were screened by PCR. Associations were analyzed using the chi-square test.

**Results:**

Overall, 106 *E. coli* isolates (30.3%) were recovered, with the highest prevalence in meat markets (39.5%) (p < 0.0001). Phylogroup A predominated (49.1%), followed by D (19.8%), B1 (18.9%), and B2 (12.3%). Virulence genes were detected in 69.8% of isolates, with *bfpA* being the most prevalent (26.4%). Universal resistance to penicillin G and erythromycin was observed, while high resistance rates were noted for ampicillin (66.0%) and tetracycline (35.8%). MDR was identified in 46.2% of isolates and was significantly more common in meat markets (p = 0.0225). The highest MDR prevalence occurred in phylogroup D (57.1%). The most frequently detected resistance genes were *bla*_SHV_, *ereA*, and *tetA*.

**Conclusion:**

Houseflies from food markets in Northern Thailand harbor virulent and MDR *E. coli*, highlighting their role as environmental sentinels and potential disseminators of antimicrobial resistance. These findings underscore the need for improved market hygiene and incorporation of insect vectors into One Health–based antimicrobial resistance surveillance strategies.

## INTRODUCTION

Antimicrobial resistance (AMR) has emerged as a critical global public health challenge and has received considerable attention from the World Health Organization (WHO) [1]. The increasing prevalence of pathogens exhibiting multidrug-resistant (MDR) phenotypes represents a major threat, as it substantially complicates the management of common infections and contributes to elevated morbidity, mortality, and healthcare costs [2, 3]. Owing to its far-reaching consequences for public health and global economic stability, the WHO has identified AMR as a strategic priority within the framework of the Sustainable Development Goals [4, 5]. Effective mitigation of AMR requires integrated approaches, including the establishment of robust surveillance systems, the promotion of targeted research, and the implementation of evidence-based public health interventions. *Escherichia coli* is a Gram-negative bacillus that forms part of the normal gastrointestinal microbiota of humans and other warm-blooded animals; however, pathogenic strains are capable of causing gastrointestinal disease as well as extraintestinal infections, such as urinary tract infections and septicemia [6]. The rising prevalence of MDR *E. coli* is therefore a major public health concern, as it restricts therapeutic options and increases the likelihood of treatment failure [7].

Houseflies are recognized mechanical vectors of a wide range of pathogens, including viruses, fungi, and bacteria such as *E. coli*. These insects can acquire and disseminate microorganisms throughout their life cycle, particularly in environments with inadequate sanitation [8]. Their feeding and breeding activities are closely linked to food markets, animal production systems, and waste disposal sites, where they can mechanically transfer pathogenic and AMR bacteria from contaminated substrates to food and food-contact surfaces [9]. Poor hygiene conditions facilitate housefly proliferation, thereby increasing the risk of pathogen transmission to humans [10]. The role of houseflies in the dissemination of AMR bacteria underscores their epidemiological importance and highlights the need for surveillance of MDR *E. coli* in food market environments, where the risk of human exposure is particularly high.

Despite the growing recognition of AMR as a major global health threat, surveillance efforts have largely focused on clinical, livestock, and food samples, with comparatively limited attention given to environmental vectors operating at the human–food interface. In particular, the role of houseflies as mechanical carriers of MDR *E. coli* in fresh-food market environments remains insufficiently characterized, especially in Northern Thailand. Existing studies from Thailand and neighboring regions have primarily reported prevalence and phenotypic antimicrobial susceptibility, with limited integration of phylogenetic background, virulence determinants, and resistance gene profiles within the same analytical framework. Moreover, comparative data across different market types (meat, vegetable, and cooked-food markets) are scarce, constraining risk assessment of differential exposure scenarios. The absence of region-specific baseline data that simultaneously link MDR phenotypes, virulence-associated genes, and phylogenetic distribution of *E. coli* in housefly populations represents a critical knowledge gap for environmental AMR surveillance and One Health–oriented interventions.

This study aimed to determine the prevalence of *E. coli* isolated from houseflies collected at meat, vegetable, and cooked-food markets in Muang District, Phayao Province, Northern Thailand, and to characterize their phylogenetic distribution, virulence gene profiles, antimicrobial resistance patterns, and MDR status. In addition, this study sought to identify resistance-associated genes and to evaluate the associations between market type, phylogenetic group, virulence carriage, and MDR phenotypes. By generating integrated baseline data from a high-risk food market setting, the study aims to contribute evidence to support AMR surveillance and control strategies within a One Health framework.

## MATERIALS AND METHODS

### Ethical approval

This study did not require formal ethical approval from an Institutional Animal Ethics Committee or Institutional Review Board, as it exclusively involved the collection of houseflies (*Musca domestica*), which are invertebrates and not subject to animal welfare regulations under international guidelines (e.g., those from the World Organization for Animal Health or local Thai regulations). No vertebrate animals, human participants, or procedures causing pain or distress were used. The study was conducted in accordance with general principles of humane insect collection and biosafety protocols at the University of Phayao. This study on *M. domestica* was reviewed and approved by the Institutional Animal Care and Use Committee of the University of Phayao, Thailand (Ethical Approval No. 640104039).

### Study period and location

This cross-sectional surveillance study was conducted from June to November 2023 in urban and peri-urban market settings in Muang District, Phayao Province, Northern Thailand.

### Sample collection

Housefly samples were collected from local meat, vegetable, and cooked-food markets selected based on public accessibility, active food-handling activities, and their relevance as high-contact environments with potential environmental contamination and human exposure.

Sampling intensity was adjusted according to market size, with five traps deployed per sampling event in cooked-food markets (small), ten in vegetable markets (medium), and fifteen in meat markets (large). In total, 350 houseflies were collected using the sticky trap method, as previously described [11]. Sterile adhesive strips were placed within market areas, and captured houseflies were transferred individually into sterile containers using forceps. Samples were transported to the laboratory and euthanized at −20°C for 1–2 h. Morphological identification was performed using a stereomicroscope to confirm species identity as *M. domestica* [12]. A single housefly was then placed in a test tube containing 2 mL of alkaline peptone water (APW) medium (HiMedia Laboratories Pvt. Ltd., Mumbai, India, Cat. No. M618-500G), vortexed, and incubated at 37°C for 16–18 h for whole-fly enrichment without separation of external and internal compartments.

### Isolation of *E. coli*

A sterile loopful of enriched APW culture was streaked onto eosin methylene blue agar (HiMedia Laboratories Pvt. Ltd., Cat. No.M317-500G) and incubated at 37°C for 24 h. Presumptive *E. coli* colonies exhibiting a metallic sheen with a dark center were selected and subjected to biochemical and morphological characterization, including Gram staining, sugar fermentation, methyl red, Voges–Proskauer, indole, and citrate tests, as previously described [13]. Confirmed colonies were cultured and stored on nutrient agar (NA) medium (HiMedia Laboratories Pvt. Ltd., Cat. No. M001-500G) and incubated at 37°C for 24 h for further analyses.

### DNA extraction

Genomic DNA was extracted using the boiling method, as previously described [14]. A single colony of *E. coli* grown on NA medium was suspended in 100 μL of sterile water in a 1.5 mL microcentrifuge tube. Samples were heated at 100°C for 10 min, cooled on ice for 5 min, and centrifuged at 10,000 rpm for 10 min. The supernatant was transferred to a new microcentrifuge tube and stored at −20°C until use.

### Molecular confirmation and phylogenetic grouping

All isolates were molecularly confirmed as *E. coli* by polymerase chain reaction (PCR) targeting the *uidA* (β-glucuronidase) gene, following Heijnen and Medema [15] with minor modifications. PCR reactions (25 μL) were prepared using OnePCR™ Ultra (Bio-Helix Co., Ltd., Keelung, Taiwan, Cat. No. 1BHC-MBA01-0100), *uidA*-F/*uidA*-R primers (Supplementary Table 1), DNA template, and sterile water. PCR products were analyzed by agarose gel electrophoresis and visualized under UV light using a gel documentation system (BIS 303 PC; DNR Bio-Imaging Systems Ltd., Jerusalem, Israel). A 100 bp DNA marker (OmniMARK 100 RTU., Bio-Helix Co., Ltd., Cat. No. DM101-0100) was used for size estimation. Amplicons were sequenced by Macrogen Inc. (Seoul, South Korea) and analyzed using BLAST in the NCBI GenBank database.

Phylogenetic grouping was performed by multiplex PCR targeting the *chuA* and *yjaA* genes and the DNA fragment *TSPE4.C2*, as described by Clermont *et al*. [16]. PCR conditions and downstream analyses followed the same procedures as described above.

### Detection of virulence genes

Virulence genes associated with diarrheagenic *E. coli* were detected by PCR using primers and conditions described previously [7]. The target genes included *aggR*, *stx1*, *stx2*, *astA*, *estp*, *esth*, *elt*, *bfpA*, *eae*, and *invE* (Supplementary Table 1).

### Antimicrobial susceptibility testing

Antimicrobial susceptibility was evaluated using the disk diffusion method in accordance with Clinical and Laboratory Standards Institute guidelines (CLSI M100-S23, 2018). Twelve antibiotics representing eight antimicrobial classes were tested: aminoglycosides (gentamicin, streptomycin), penicillins/β-lactams (penicillin G, ampicillin), carbapenems (imipenem, meropenem), tetracyclines (tetracycline, tigecycline), phenicols (chloramphenicol), fluoroquinolones (ciprofloxacin), macrolides (erythromycin), and sulfonamides (trimethoprim–sulfamethoxazole) (HiMedia Laboratories Pvt. Ltd., Cat. No. SD170, SD031, SD028, SD002, SD073, SD727, SD037, SD278, SD006, SD060, SD013, and SD010, respectively). Mueller–Hinton agar plates (HiMedia Laboratories Pvt. Ltd., Cat. No. M173) were inoculated with a 0.5 McFarland standardized suspension and incubated at 37°C for 18–24 h. Inhibition zones were measured in millimeters. *E. coli* TISTR 073 served as the quality control strain. Isolates resistant to one or more antibiotics in three or more antimicrobial classes were classified as MDR [17, 18].

### Detection of antimicrobial resistance genes

Phenotypically resistant *E. coli* isolates were screened for resistance-associated genes by PCR. Target genes included *acc(3)-IV*, *aac(6)-Ib-cr*, *aadA1*, *CITM*, *bla*_SHV_, *catA1*, *ereA*, *sul1*, and *tetA*. Primer sequences and expected amplicon sizes are provided in Supplementary Table 1. PCR conditions followed previously described protocols [7, 10, 15, 16, 19, 20].

### Statistical analysis

Descriptive data are presented as frequencies and percentages. Differences in categorical variables, including *E. coli* prevalence, virulence gene positivity, MDR phenotypes, and AMR gene distribution among market types, were assessed using the chi-square test. Associations between phylogenetic groups and virulence status, as well as between phylogenetic groups and MDR status, were also evaluated. Statistical analyses were performed using GraphPad Prism version 5.00 for Windows (GraphPad Software Inc., San Diego, CA, USA), and p ≤ 0.05 was considered statistically significant.

## RESULTS

### Prevalence and phylogroup distribution of *E. coli* isolates

In this study, 106 (30.3%) *E. coli* strains were isolated from 350 housefly samples and confirmed by PCR analysis. The highest prevalence was observed in the meat market (39.5%), followed by the cooked-food market (26.3%) and the vegetable market (14.0%) (Table 1). A statistically significant difference in *E. coli* prevalence was detected among market types (p < 0.0001). Phylogenetic analysis revealed that phylogroup A was predominant (52 isolates; 49.1%), followed by phylogroups D (21 isolates; 19.8%), B1 (20 isolates; 18.9%), and B2 (13 isolates; 12.3%) (Table 1).

**Table 1:** Prevalence and occurrence rate of phylogenetic groups of *Escherichia coli* isolates obtained from houseflies at various markets in Phayao Province, Northern Thailand.

Sampling area	Number of samples collected	*E. coli*-positive samples, n (%)	A, n (%)	B1, n (%)	B2, n (%)	D, n (%)
Meat market	200	79 (39.5)	42 (53.2)	11 (13.9)	7 (8.9)	19 (24.1)
Vegetable market	100	14 (14.0)	7 (50.0)	5 (35.7)	0 (0.0)	2 (14.3)
Cooked-food market	50	13 (26.3)	3 (23.1)	4 (30.8)	6 (46.2)	0 (0.0)
Total	350	106 (30.3)	52 (49.1)	20 (18.9)	13 (12.3)	21 (19.8)

n = Number of isolates, % = Percentage within *E. coli*-positive samples, A, B1, B2, and D = Phylogenetic groups of *E. coli*. p-value: <0.0001.

### Distribution of virulence genes in *E. coli* isolates

All *E. coli* isolates were screened for 10 virulence genes associated with diarrheagenic pathotypes. Overall, 74 isolates (69.8%) were positive for at least one virulence gene (Table 2). The most prevalent gene was *bfpA* (26.4%; 28/106), followed by *astA* (12.3%; 13/106), *stx1* (7.5%; 8/106), *esth* (4.7%; 5/106), *eae* (2.8%; 3/106), and *estp* (0.9%; 1/106). In contrast, *elt*, *invE*, and *stx2* were not detected. No significant difference was observed in the distribution of virulence gene-positive isolates among market types (p = 0.057). Isolates carrying two virulence genes accounted for 15.1% (16/106), with four observed combinations, the most common being *aggR*–*astA* (9.4%; 10/106), followed by *aggR*–*bfpA* (3.8%; 4/106), *astA*–*esth* (0.9%; 1/106), and *bfpA*–*stx* (0.9%; 1/106).

**Table 2:** Virulence gene profiles of *Escherichia coli* isolated from houseflies in various markets in Phayao Province, Northern Thailand.

Virulence profiles	Meat market (n = 79)	Vegetable market (n = 14)	Cooked-food market (n = 13)	Total (n = 106)	p-value
Negative	24 (30.4)	7 (50.0)	1 (7.7)	32 (30.2)	0.0570
Positive virulence	55 (69.6)	7 (50.0)	12 (92.3)	74 (69.8)	
One gene					
*astA*	13 (16.5)	0 (0.0)	0 (0.0)	13 (12.3)	
*bfpA*	24 (30.4)	1 (7.1)	3 (23.1)	28 (26.4)	
*eae*	3 (3.8)	0 (0.0)	0 (0.0)	3 (2.8)	
*esth*	5 (6.3)	0 (0.0)	0 (0.0)	5 (4.7)	
*estp*	0 (0.0)	0 (0.0)	1 (7.7)	1 (0.9)	
*stx1*	4 (5.1)	4 (28.6)	0 (0.0)	8 (7.5)	
Two genes					
*aggR–astA*	5 (6.3)	1 (7.1)	4 (30.8)	10 (9.4)	
*aggR–bfpA*	0 (0.0)	0 (0.0)	4 (30.8)	4 (3.8)	
*astA–esth*	1 (1.3)	0 (0.0)	0 (0.0)	1 (0.9)	
*bfpA–stx*	0 (0.0)	1 (7.1)	0 (0.0)	1 (0.9)	

n = Number of *Escherichia coli* isolates, % = Percentage within each market group, *astA* = enteroaggregative heat-stable toxin gene, *bfpA* = bundle-forming pilus gene, *eae* = intimin gene, *esth* = heat-stable enterotoxin gene (human type), *estp* = heat-stable enterotoxin gene (porcine type), *stx1* = Shiga toxin 1 gene, *aggR* = transcriptional activator gene. p-values were calculated using the chi-square test for comparisons of the distribution of virulence gene-positive *E. coli* isolates among market types.

### Phylogenetic distribution of virulence-associated gene profiles

Virulence gene profiles varied across phylogenetic groups (Supplementary Table 2). Phylogroup A exhibited the greatest diversity of virulence-associated genes, with *bfpA* being the most prevalent profile (36.4%; 12/33), whereas rare profiles such as *bfpA*–*stx* were least frequent (3.0%; 1/33). In phylogroup B1, *bfpA* predominated (35.7%; 5/14), while *stx1* occurred at the lowest frequency (7.1%; 1/14). Phylogroup B2 showed marked dominance of *bfpA* (63.6%; 7/11), representing the highest single-gene prevalence among all groups, whereas *estp* was detected infrequently and exclusively in this group (9.1%; 1/11). Phylogroup D displayed a more even distribution of virulence profiles, with *bfpA*, *astA*, *stx1*, and *aggR*–*astA* representing the most common patterns (18.8%–25.0%; 3–4/16), while *eae* was the least prevalent (6.3%; 1/16). No significant association was found between phylogenetic group and overall virulence gene positivity (p = 0.4314).

### Antibiotic resistance phenotype of *E. coli* isolates

Phenotypic antimicrobial resistance was widely observed among *E. coli* isolates from all markets. All 106 isolates were tested against 12 antibiotics representing eight antimicrobial classes. Complete resistance to penicillin G (100%) and erythromycin (100%) was observed in all isolates (Table 3). High resistance rates were recorded for ampicillin (66.0%; 70/106), tetracycline (35.8%; 38/106), and chloramphenicol (17.0%; 18/106). Lower resistance rates were noted for gentamicin (11.3%; 12/106), streptomycin (11.3%; 12/106), sulfonamide (11.3%; 12/106), and ciprofloxacin (2.8%; 3/106). No resistance was detected to imipenem, meropenem, or tigecycline.

**Table 3:** Antibiotic resistance profile of *Escherichia coli* isolates.

Drug classes	Antibiotics	Meat (n = 79)	Vegetable market (n = 14)	Cooked-food market (n = 13)	Total (n = 106)
Aminoglycosides	Gentamicin	7 (8.9)	0 (0.0)	5 (38.5)	12 (11.3)
	Streptomycin	12 (15.2)	0 (0.0)	0 (0.0)	12 (11.3)
Penicillin	Penicillin G	79 (100.0)	14 (100.0)	13 (100.0)	106 (100.0)
	Ampicillin	55 (69.9)	7 (50.0)	8 (61.5)	70 (66.0)
Carbapenem	Imipenem	0 (0.0)	0 (0.0)	0 (0.0)	0 (0.0)
	Meropenem	0 (0.0)	0 (0.0)	0 (0.0)	0 (0.0)
Tetracycline	Tetracycline	33 (41.8)	0 (0.0)	5 (38.5)	38 (35.8)
	Tigecycline	0 (0.0)	0 (0.0)	0 (0.0)	0 (0.0)
Phenicol	Chloramphenicol	13 (16.5)	0 (0.0)	5 (38.5)	18 (17.0)
Fluoroquinolone	Ciprofloxacin	3 (3.8)	0 (0.0)	0 (0.0)	3 (2.8)
Macrolide	Erythromycin	79 (100.0)	14 (100.0)	13 (100.0)	106 (100.0)
Sulfonamides	Trimethoprim/sulfamethoxazole	10 (12.7)	2 (14.3)	0 (0.0)	12 (11.3)

Multidrug resistance was identified in 46.2% (49/106) of isolates (Table 4). The prevalence of MDR isolates was significantly higher in the meat market than in the vegetable and cooked-food markets (p = 0.0225). A total of 23 distinct AMR patterns were observed, with ampicillin–erythromycin–penicillin–tetracycline being the most frequent MDR profile (11.3%; 12/106). Only two isolates, both from the meat market, exhibited resistance to seven tested antibiotics.

**Table 4:** Antibiotic resistance patterns of *Escherichia coli* isolates.

Antibiotic-resistant patterns	Meat (n = 79)	Vegetable market (n = 14)	Cooked-food market (n = 13)	Total (n = 106)	p-value
MDR	42 (53.2)	2 (14.3)	5 (38.5)	49 (46.2)	0.0225
Two drugs					
E–P	17 (21.5)	7 (50.0)	5 (38.5)	29 (27.4)	
Three drugs					
AMP–E–P	20 (25.3)	5 (35.7)	3 (23.1)	28 (26.4)	
E–P–S	1 (1.3)	0 (0.0)	0 (0.0)	1 (0.9)	
E–P–TE	2 (2.5)	0 (0.0)	0 (0.0)	2 (1.9)	
E–P–GEN	1 (1.3)	0 (0.0)	0 (0.0)	1 (0.9)	
E–P–C	1 (1.3)	0 (0.0)	0 (0.0)	1 (0.9)	
Four drugs					
AMP–E–P–TE	12 (15.2)	0 (0.0)	0 (0.0)	12 (11.3)	
AMP–E–P–SXT	1 (1.3)	2 (14.3)	0 (0.0)	3 (2.8)	
AMP–E–P–GEN	1 (1.3)	0 (0.0)	0 (0.0)	1 (0.9)	
AMP–E–P–S	1 (1.3)	0 (0.0)	0 (0.0)	1 (0.9)	
E–P–TE–C	1 (1.3)	0 (0.0)	0 (0.0)	1 (0.9)	
Five drugs					
AMP–E–P–TE–SXT	2 (2.5)	0 (0.0)	0 (0.0)	2 (1.9)	
AMP–E–P–TE–S	5 (6.3)	0 (0.0)	0 (0.0)	5 (4.7)	
AMP–E–P–TE–C	1 (1.3)	0 (0.0)	0 (0.0)	1 (0.9)	
AMP–E–P–S–SXT	1 (1.3)	0 (0.0)	0 (0.0)	1 (0.9)	
E–P–TE–SXT	1 (1.3)	0 (0.0)	0 (0.0)	1 (0.9)	
Six drugs					
AMP–E–P–TE–C–GEN	4 (5.1)	0 (0.0)	5 (38.5)	9 (8.5)	
AMP–E–P–C–SXT–CIP	2 (2.5)	0 (0.0)	0 (0.0)	2 (1.9)	
AMP–E–P–TE–S–C	1 (1.3)	0 (0.0)	0 (0.0)	1 (0.9)	
AMP–E–P–TE–S–GEN	1 (1.3)	0 (0.0)	0 (0.0)	1 (0.9)	
AMP–E–P–TE–S–SXT	1 (1.3)	0 (0.0)	0 (0.0)	1 (0.9)	
Seven drugs					
AMP–E–P–TE–C–SXT–CIP	1 (1.3)	0 (0.0)	0 (0.0)	1 (0.9)	
AMP–E–P–TE–C–SXT–S	1 (1.3)	0 (0.0)	0 (0.0)	1 (0.9)	

MDR = Multidrug resistance, AMP = Ampicillin, E = Erythromycin, P = Penicillin G, TE = Tetracycline, S = Streptomycin, GEN = Gentamicin, C = Chloramphenicol, CIP = Ciprofloxacin, SXT = Trimethoprim/sulfamethoxazole, n = Number of *Escherichia coli* isolates, % = Percentage within each market group. p-values were calculated using the chi-square test to compare the distribution of MDR phenotypes among market types.

### Distribution of MDR across phylogenetic groups and market types

Among the 106 *E. coli* isolates, MDR positivity was highest in phylogroup D (57.1%; 12/21), followed by B1 (50.0%; 10/20) and A (48.1%; 25/52), and lowest in phylogroup B2 (15.4%; 2/13) (Supplementary Table 3). Most MDR-positive isolates originated from the meat market (85.7%; 42/49), followed by the cooked-food market (10.2%; 5/49) and the vegetable market (4.1%; 2/49). By phylogenetic group, MDR-positive isolates in groups A and D were almost exclusively associated with the meat market, whereas group B1 included isolates from all three market types and group B2 from meat and cooked-food markets only. No significant association was detected between phylogenetic group and overall MDR positivity (p = 0.1037).

### Antibiotic resistance gene profiles of *E. coli* isolates

**Figure 1:**
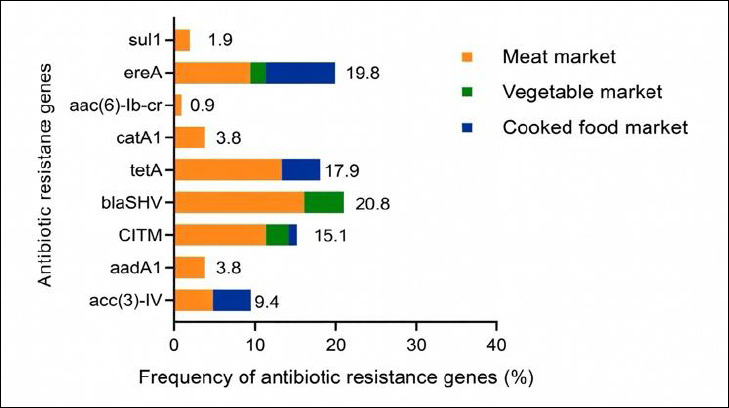
Resistance gene profiles were investigated among *Escherichia coli* strains obtained from houseflies in different markets. *sul1* (sulfonamide), *ereA* (erythromycin), *aac(6)-Ib-cr* (ciprofloxacin), *catA1* (chloramphenicol), *tetA* (tetracycline), *bla*_SHV_ (penicillin), *CITM* (ampicillin), *aadA1* (streptomycin), *acc(3)-IV* (gentamicin).

The distribution of AMR genes among resistant *E. coli* isolates by market type is shown in Figure 1 and Supplementary Table 4. The most prevalent gene was *bla*_SHV_ (20.8%), followed by *ereA* (19.8%), *tetA* (17.9%), *CITM* (15.1%), *acc(3)-IV* (9.4%), *catA1* (3.8%), *aadA1* (3.8%), *sul1* (1.9%), and *aac(6)-Ib-cr* (0.9%). The majority of AMR gene–harboring isolates originated from meat markets.

Overall, 67 (63.2%) of the 106 isolates carried at least one AMR gene (Table 5). The most frequently detected single gene was *bla*_SHV_ (12.3%), followed by *ereA* (11.3%) and *CITM* (8.5%). Among isolates carrying multiple resistance genes, the most common combinations were *acc(3)-IV–tetA* (4.7%) and *acc(3)-IV–ereA–tetA* (4.7%). A significant difference was observed in the distribution of AMR-positive isolates among market types (p = 0.0394).

**Table 5:** Antibiotic resistance gene patterns of *Escherichia coli* strains obtained from houseflies in different markets in Phayao Province, Northern Thailand.

Antibiotic resistance gene expression patterns	Meat (n = 79)	Vegetable Market (n = 14)	Cooked-food market (n = 13)	Total (n = 106)	p-value
Negative	34 (43.0)	4 (25.6)	1 (7.7)	39 (36.8)	0.0394
Positive resistance genes	45 (57.0)	10 (97.4)	12 (92.3)	67 (63.2)	
One gene					
*aac(6)-Ib-cr*	1 (1.3)	0 (0.0)	0 (0.0)	1 (0.9)	
*aadA1*	2 (2.5)	0 (0.0)	0 (0.0)	2 (1.9)	
*bla*_SHV_	8 (10.1)	5 (35.7)	0 (0.0)	13 (12.3)	
*catA1*	3 (3.8)	0 (0.0)	0 (0.0)	3 (2.8)	
*CITM*	5 (6.3)	3 (21.4)	1 (7.7)	9 (8.5)	
*ereA*	4 (5.1)	2 (14.3)	6 (46.2)	12 (11.3)	
*tetA*	6 (7.6)	0 (0.0)	0 (0.0)	6 (5.7)	
Two genes					
*acc(3)-IV–tetA*	3 (3.8)	0 (0.0)	2 (15.4)	5 (4.7)	
*bla*_SHV_*–CITM*	4 (5.1)	0 (0.0)	0 (0.0)	4 (3.8)	
*CITM–ereA*	2 (2.5)	0 (0.0)	0 (0.0)	2 (1.9)	
Three genes					
*aadA1– bla*_SHV_*–tetA*	2 (2.5)	0 (0.0)	0 (0.0)	2 (1.9)	
*acc(3)-IV–ereA–tetA*	2 (2.5)	0 (0.0)	3 (21.3)	5 (4.7)	
*bla*_SHV_*–CITM–ereA*	1 (1.3)	0 (0.0)	0 (0.0)	1 (0.9)	
*bla*_SHV_*–ereA–sul1*	1 (1.3)	0 (0.0)	0 (0.0)	1 (0.9)	
Four genes					
*bla*_SHV_*–catA1–sul1–tetA*	1 (1.3)	0 (0.0)	0 (0.0)	1 (0.9)	

n = Number of *E. coli* isolates, % = Percentage within each market group, *aac(6)-Ib-cr* = Ciprofloxacin resistance gene, *aadA1* = Streptomycin resistance gene, *acc(3)-IV* = Gentamicin resistance gene, *bla*_SHV_ = Penicillin resistance gene, *catA1* = Chloramphenicol resistance gene, *CITM* = Ampicillin resistance gene, *ereA* = Erythromycin resistance gene, *sul1* = Sulfonamide resistance gene, *tetA* = Tetracycline resistance gene.

p-values were calculated using the chi-square test for comparisons of AMR gene-positive *E. coli* isolate distribution among market types.

## DISCUSSION

### Role of houseflies in pathogen transmission

Houseflies (*M. domestica*) are among the most common synanthropic insects coexisting with human communities, where they cause nuisance and contribute to public health problems. High housefly densities are often indicative of poor household and environmental hygiene [21]. Houseflies possess a strong capacity to acquire and disseminate pathogens from septic sources to other environments through contamination of their body surfaces, including feet, wings, and mouthparts, thereby acting as mechanical vectors for pathogenic agents and AMR dissemination [22].

### Prevalence of *E. coli* in market-associated houseflies

In this study, *E. coli* was isolated from houseflies collected from meat, vegetable, and cooked-food markets in Muang District, Phayao Province, Northern Thailand. The overall prevalence of *E. coli* was 30.3%, with the highest prevalence observed in meat markets (39.5%). These findings are consistent with reports from India, where a comparable prevalence of 30% was observed [23], but higher than that reported from Bangladesh (19%) [24]. In contrast, substantially higher contamination rates ranging from 51.4% to 77% have been documented in other geographic regions [8, 10, 25]. The elevated prevalence observed in meat markets may be attributed to inadequate sanitation and frequent exposure of flies to animal feces and contaminated materials during meat handling and processing [26, 27].

### Phylogenetic distribution of *E. coli* isolates

Phylogenetic analysis revealed that phylogroup A predominated among the *E. coli* isolates (49.1%), consistent with previous studies identifying phylogroup A as the most prevalent group across diverse sources [28]. This phylogroup is commonly associated with commensal as well as pathogenic *E. coli* strains in humans and animals. However, the proportion observed in the present study was slightly higher than the approximately 40% prevalence reported in similar investigations conducted in other regions [29].

### Virulence gene carriage and pathogenic potential

Virulence gene detection is essential for assessing the pathogenic potential of *E. coli*, particularly diarrheagenic strains [30]. In the present study, 69.8% of isolates carried at least one virulence gene, indicating substantial pathogenic potential among housefly-associated *E. coli*. The most frequently detected gene was *bfpA* (26.4%), which occurred predominantly in isolates from meat markets. This prevalence exceeds that reported from surface water in Australia (24%) [31]. The *bfpA* gene is a hallmark of EPEC and encodes bundle-forming pili that mediate adherence to intestinal epithelial cells, leading to diarrheal disease [32].

The *stx1* gene, associated with EHEC, was detected in 7.5% of isolates, a prevalence slightly lower than that reported by Sobur *et al*. [33]. The absence of *invE* and *elt*, which are associated with EIEC and ETEC, respectively [34], may reflect the low circulation of these pathotypes in the studied setting. The lack of significant differences in virulence gene distribution among market types (p = 0.057) highlights the need for continued surveillance of DEC.

### Phylogenetic spread of diarrheagenic virulence markers

The detection of multiple DEC-associated virulence markers (*eae*, *bfpA*, *aggR*, *astA*, *stx1*, *esth*, and *estp*) across different phylogenetic groups, with *bfpA* predominating, suggests that these virulence traits are not restricted to specific phylogroups. This distribution aligns with previous observations of genetic heterogeneity among atypical EPEC strains and supports the concept that DEC virulence determinants can spread via horizontal gene transfer, thereby weakening strict phylogroup–virulence associations [34–36].

### Antimicrobial resistance patterns in *E. coli* isolates

The detection of antibiotic-resistant *E. coli* in houseflies represents a significant public health concern. All isolates exhibited complete resistance to penicillin G and erythromycin, consistent with previous reports documenting high resistance rates to these antimicrobials among housefly-associated *E. coli* [8, 10, 37, 38]. Elevated resistance to ampicillin (66.0%), tetracycline (35.8%), and chloramphenicol (17.0%) was observed, exceeding rates reported from Bangladesh, Iran, and Nigeria [10, 22, 39]. Conversely, the absence of resistance to imipenem and meropenem corroborates findings from China, indicating sustained efficacy of carbapenems against *E. coli* [25].

### Multidrug resistance and phylogenetic associations

MDR was detected in 46.2% of isolates, with a significantly higher prevalence in meat markets than in other market types, reflecting environmental contamination and antimicrobial exposure [40]. Comparable MDR rates have been reported in urban environments [38], whereas lower rates were observed in dairy farm settings [41]. In contrast, higher MDR prevalence has been documented in hospital-adjacent environments [35]. The most frequent MDR pattern, ampicillin–erythromycin–penicillin–tetracycline, underscores the adaptability of *E. coli* to commonly used antibiotics [41]. Although no significant association was found between phylogenetic group and MDR phenotype (p = 0.1037), MDR is largely driven by mobile genetic elements rather than phylogenetic background alone [42–44].

### Antimicrobial resistance gene profiles and public health implications

AMR gene carriage was detected in 63.2% of isolates, highlighting the role of houseflies as environmental reservoirs of resistance determinants. The most prevalent gene was *bla*_SHV_ (20.8%), although this rate was lower than those reported in food centers and fish markets [10, 40]. Moderate prevalence of *ereA*, *tetA*, and *CITM* further supports the linkage between phenotypic resistance and underlying genetic mechanisms. Variability in gene prevalence across studies likely reflects geographic and environmental differences [41, 45]. These findings emphasize the importance of sustained surveillance and targeted interventions to limit environmental dissemination of AMR genes.

## CONCLUSION

This study demonstrated that *E. coli* is commonly carried by *M. domestica* in fresh-food market environments, with an overall prevalence of 30.3%. The highest contamination and MDR burden were consistently observed in meat markets. Phylogroup A predominated, while phylogroup D contributed disproportionately to MDR positivity. A high proportion of isolates (69.8%) harbored at least one DEC-associated virulence gene, with *bfpA* being the most prevalent, indicating a notable presence of EPEC-linked determinants. Phenotypic resistance was widespread, with universal resistance to penicillin G and erythromycin and an overall MDR prevalence of 46.2%. Moreover, 63.2% of isolates carried at least one AMR gene, with *bla*_SHV_, *ereA*, and *tetA* being the most frequently detected.

These findings highlight fresh-food markets, particularly meat markets, as critical interfaces for environmental dissemination of virulent and MDR *E. coli*. The detection of DEC-associated virulence markers and AMR genes in houseflies underscores the importance of incorporating insect vectors into routine AMR surveillance programs. Improved market hygiene, effective waste management, and fly control strategies are essential to reduce the risk of food contamination and subsequent human exposure within a One Health framework.

A key strength of this study lies in its integrated approach, combining prevalence estimation, phylogenetic grouping, virulence profiling, phenotypic resistance testing, and AMR gene detection within the same isolates. The comparison across different market types provides context-specific insights into exposure risk, while the focus on houseflies expands current understanding of non-clinical AMR reservoirs.

This cross-sectional study was confined to a single province, which may limit extrapolation to other regions. The absence of whole-genome sequencing restricted resolution of clonal relationships and mobile genetic elements. In addition, antimicrobial susceptibility was assessed using disc diffusion only, without MIC determination, and direct fly-to-food transmission and human exposure were not quantitatively evaluated.

Future studies should incorporate longitudinal sampling, whole-genome sequencing, and quantitative risk assessment to better elucidate transmission pathways of MDR *E. coli*. Integrating entomological surveillance with food safety and public health monitoring will strengthen One Health–based AMR control strategies. Evaluating the effectiveness of targeted hygiene and vector control interventions in market settings is also warranted.

Overall, this study provides baseline evidence that *M. domestica* in fresh-food markets act as important environmental reservoirs of virulent and MDR *E. coli*. Addressing AMR at the food–environment–human interface requires coordinated surveillance and intervention strategies, reinforcing the critical role of environmental vectors in AMR epidemiology.

## DATA AVAILABILITY

The supplementary data can be made available from the corresponding author upon request.

## AUTHORS’ CONTRIBUTIONS

KK: Conceptualized and designed the study, collected samples, and performed the experiments. KK, SU, AS, and NS: Analyzed the data and drafted the manuscript. KK, SU, and NS: Reviewed and edited the manuscript. KK also secured funding for the study. OJ and CT: Data analysis. All authors have reviewed and approved the final manuscript of the manuscript.
